# Case report: A compound heterozygous mutations in *ARSA* associated with adult-onset metachromatic leukodystrophy

**DOI:** 10.3389/fneur.2022.1011019

**Published:** 2022-10-17

**Authors:** Bing-lei Wang, Fen-lei Lu, Yu-chen Sun, Hui-juan Wang

**Affiliations:** ^1^Department of Neurology, The Second Hospital of Hebei Medical University, Shijiazhuang, China; ^2^Department of Neurosurgery, The Second Hospital of Hebei Medical University, Shijiazhuang, China

**Keywords:** metachromatic leukodystrophy (MLD), autosomal recessive inherited, arylsulfatase A (*ARSA*), compound heterozygous mutations, case report

## Abstract

Metachromatic Leukodystrophy (MLD) is a rare autosomal recessive disease, which is caused by mutations in the arylsulfatase A (*ARSA*) gene. The *ARSA* gene is located on chromosome 22q13, containing eight exons. According to the age of onset, MLD can be divided into late infantile type, juvenile type, and adult type. Adult MLD has an insidious onset after the age of 16 years. Additionally, intellectual as well as behavioral changes, such as memory deficits or emotional instability, are commonly the first presenting symptoms. There is a study that reported an adult-onset MLD manifested cognitive impairment progressively due to compound heterozygous mutations of NM_000487: c.[185_186dupCA], p.(Asp63GlnfsTer18), and NM_000487: c.[154G>T], p.(Gly172Cys), rs74315271 in the *ARSA* gene, finding that the c.[154G>T], p.(Gly172Cys) is a novel missense mutation. Brain magnetic resonance imaging (MRI) revealed symmetrical demyelination of white matter. The activity of ARSA enzymatic in leukocytes decreased. Nerve conduction studies displayed that evidence of polyneuropathy was superimposed upon diffuse, uniform demyelinating, and sensorimotor polyneuropathy. Family genes revealed that each family member carried one of two heterozygous mutant genes. She has been discharged and is currently being followed up. This study found a compound heterozygous mutation in the *ARSA* gene associated with MLD and identified a novel missense mutation NM_000487: c.[154G>T], p.(Gly172Cys), rs74315271. This will provide a critical clue for prenatal diagnosis of the offspring in this family, and expand the mutation spectrum of MLD-related *ARSA*.

## Introduction

Metachromatic Leukodystrophy (MLD) is an autosomal recessive inherited disease caused by the deficiency of enzyme arylsulfatase A *(ARSA)*, which can convert cerebroside sulfatide, a major component of myelin, into cerebroside (1). It is estimated that the overall incidence of autosomal recessive MLD is 1:40,000–1:1,00,000 (2). The decrease or complete absence of *ARSA* activity leads to the storage of sulfatide in neurons and glial cells, causing neurodegeneration and demyelination in the central nervous systems (CNS) and peripheral nervous systems (PNS) (3). *ARSA*, the pathogenic gene of MLD, is located on chromosome 22q13 with eight exons and is transcribed into three kinds of mRNA with a total length of 3.2 kb (4). To date, ~279 MLD-relevant unique mutations have been identified in the *ARSA* gene (https://databases.lovd.nl/shared/genes/ARSA). In a few patients, MLD is caused by a deficiency of activator protein saposin B (5). According to the age at onset, MLD can be divided into late infantile type, juvenile type, and adult type (6). The clinical manifestation of late infantile MLD begins at 30 months old. This type is considered to be the most severe, which is characterized by a lack of or little residual *ARSA* activity and entails rapid neurodegeneration. Patients with infantile MLD show delayed psychomotor development, which is characterized by impairment of speech, gross, and fine motor development. Peripheral neuropathy is also observed, which is associated with decreased motor and sensory nerve conduction. The juvenile type, with an onset between 3 and 16 years old is further subdivided into the early juvenile and late juvenile depending on whether the onset is before or after 6 years old. In the juvenile type, cognitive impairment and behavioral variation are frequently observed, followed by the deterioration of central and peripheral motility and epilepsy. Adult MLD has an insidious onset after the age of 16 years. Intellectual and behavioral changes, such as memory deficits or emotional instability, are usually the first presenting symptoms (7). It is the least common of the three major clinical variants and is often mistakenly diagnosed as early-onset dementia (8) or schizophrenia (9, 10).

This paper reported a rare case of adult-onset MLD, which was caused by compound heterozygous mutations in the *ARSA* gene, and identified a novel missense mutation. This paper presented the following case in accordance with the CARE reporting checklist.

## Case presentation

**Figure 1:**
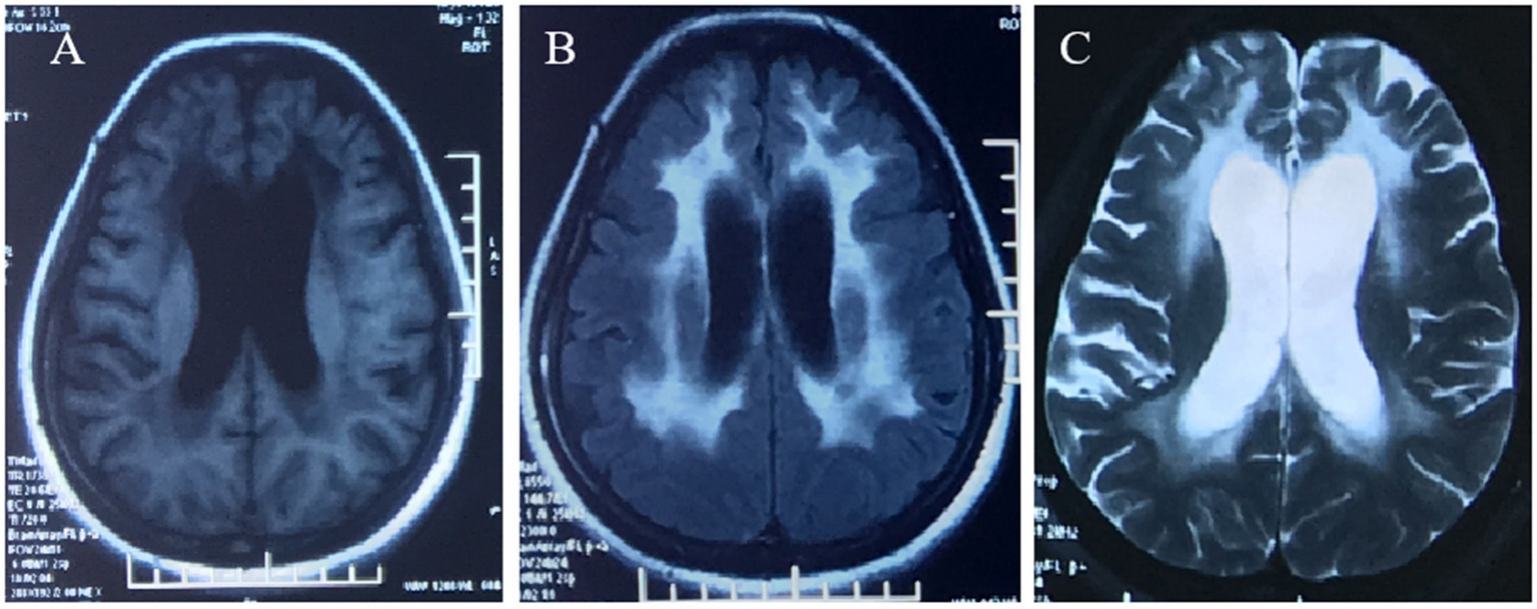
Brain MRI demonstrated diffuse, symmetrical abnormal signal in the bilateral cerebral white matter, which was low signal in T1WI **(A)**, high signal in FLAIR **(B)**, and high signal in T2WI **(C)**.

**Figure 2:**
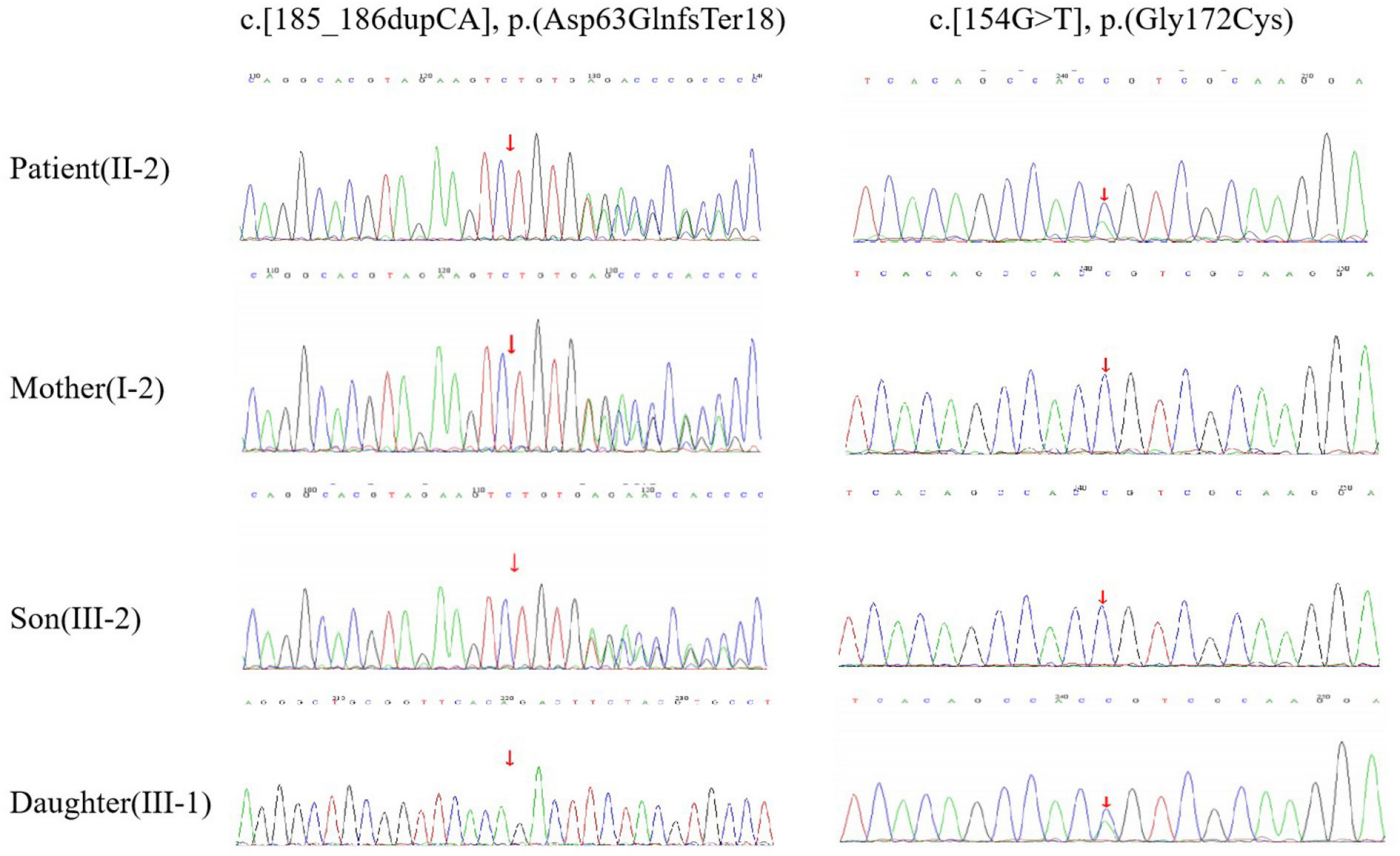
DNA sequencing. The patient (II-2) had compound heterozygous mutations of c.[185_186dupCA], p.(Asp63GlnfsTer18) and c.[154G>T], p.(Gly172Cys) in the ARSA gene. Her mother (I-2) and son (III-2) had the heterozygous mutation of c.[185_186dupCA], p.(Asp63GlnfsTer18). Her daughter (III-1) had the heterozygous mutation of c.154G>T, p.(Gly172Cys).

**Figure 3:**
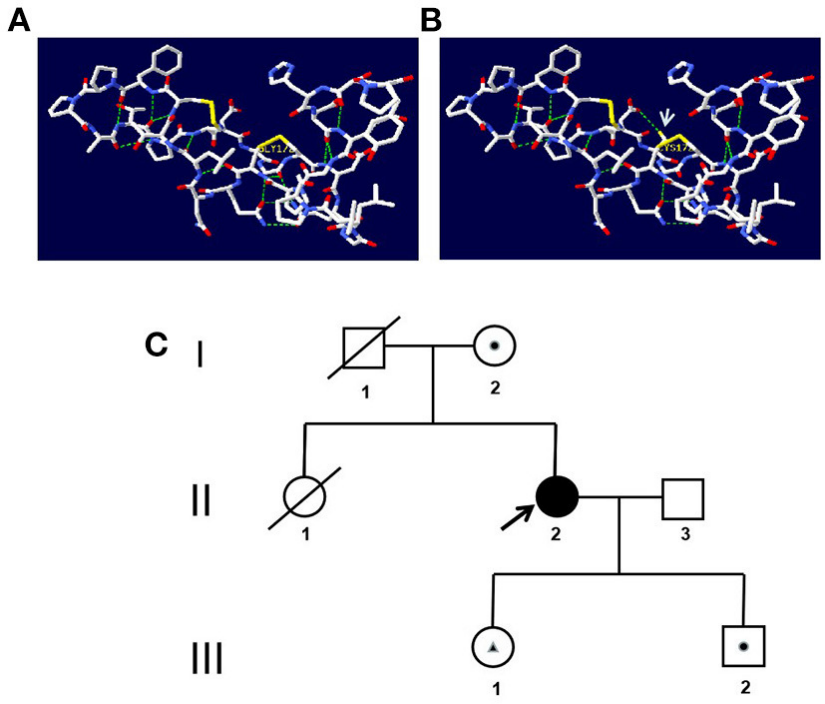
**(A,B)** Prediction of the three-dimensional structure of the protein. **(A)** The wild type of p.(Gly172Cys). **(B)** The mutated type of p.(Gly172Cys). The arrow indicates the mutation site. **(C)** Genetic family tree. A square represents a male, and a circle represents a female. A shaded symbol shows the proband, and a line across the symbol shows the deceased individual. A dot within the symbol represents carrier status for mutation c.[185_186dupCA], p.(Asp63GlnfsTer18). A triangle within the symbol represents carrier status for mutation c.[154G>T], p.(Gly172Cys).

A 42-year-old woman suffered from progressive memory loss in the first half of the year before our evaluation. She gave birth at full term *via* normal vaginal delivery without distress and dysmorphic features and grew normally. Half a year ago, she began to lose her memory, being unable to recall what just happened, and progressively aggravated. There was no known antecedent brain injury, and her medical history was not obvious. Her father died of liver cancer, and her sister who suffered from unexplained dementia finally died at the age of 40 years. She had two children, a boy and a girl, both of whom were in good health. Neurological examination displayed remarkable symptoms in reaction dullness and memory loss, as well as horizontal nystagmus. The motor system examination revealed that her muscle strength was normal (Grade 5) and her movements were coordinated. The results of the sense system examination were normal. Tendon reflexes of upper and lower extremities markedly decreased without lateralization. She graduated from junior high school with a score of 8 on the Montreal Cognitive Assessment (MoCA) and 15 on the Mini-Mental State Examination (MMSE). She was diagnosed with moderate cognitive impairment. Adult-onset, chronic progress, and high-level brain function were affected, mainly manifested as cognitive impairment. Moreover, the peripheral nerve might be involved according to the examination of weakened tendon reflexes. Neuroradiologically, brain magnetic resonance imaging (MRI) scans demonstrated diffuse and symmetrical abnormal signals in the cerebral white matter, especially around the top of the lateral ventricle (Figure 1). The activity of *ARSA* measured in white blood cells was 14.13 nmol/17h/mg, which was significantly lower than the normal value (>58 nmol/17h/mg). Nerve conduction studies showed that the evidence of polyneuropathy was superimposed upon diffuse, uniform demyelinating, and sensorimotor polyneuropathy. Electromyography (EMG) was remarkable for fibrillations and positive waves were limited to the right musculi abductor pollicis brevis. Genetic analysis indicated that there were two heterozygous mutations in the exon region of the *ARSA* gene: (1) NM_000487: c.[185_186dupCA], p.(Asp63GlnfsTer18). A duplication of CA nucleotides located in exon 1 at c.185_186 resulted in a frameshift mutation (Figure 2). (2) NM_000487: c.[154G>T], p.(Gly172Cys), rs74315271. A missense mutation of *ARSA* in exon 3 resulted in guanine being changed into thymine at nucleotide 154 (Figure 2), and amino acid Gly being replaced by Cys (Figures 3A,B). C.[185_186dupCA], p.(Asp63GlnfsTer18) was previously reported as a pathogenic mutation in the *ARSA* gene associated with MLD, but c.[154G>T], p.(Gly172Cys) was a novel mutation, which has not been reported in exome analysis. To predict whether this novel mutation is deleterious or not, the function of protein was predicted. Rare Exome Variant Ensemble Learner (REVEL), Polymorphism Phenotyping v2 (PolyPhen-2), MutationTaster, and Genomic Evolutionary Rate Profiling+ (GERP+) all indicated that the mutation was deleterious. She was eventually diagnosed with adult-onset MLD. Family genetic analysis revealed that her mother and son were identified to carry the heterozygous mutation of c.[185_186dupCA], p.(Asp63GlnfsTer18), and her daughter was the carrier of the heterozygous mutation of c.[154G>T], p.(Gly172Cys) (Figure 2). Unfortunately, this study could not collect blood samples from her father and sister due to the fact that they had passed away. The mode of inheritance of MLD is autosomal recessive, and a genetic family tree (Figure 3C) had been made. The patient was discharged soon after admission. At present, the patient is currently under follow-up.

All procedures performed in research involving human subjects were in accordance with the Helsinki Declaration (revised in 2013), and this study was approved by the Ethics Committee of the Second Hospital of Hebei Medical University (Approval Letter No. 2022-P024).

## Discussion

Metachromatic Leukodystrophy is a kind of autosomal recessive inherited lysosomal disorder due to the deficiency of the *ARSA* enzyme, which is also the most frequently encountered by Chinese patients (11). MLD is usually classified into late infantile type, juvenile type, and adult type according to the age of onset. Adult MLD is the less severe and the least common type of this disease, which is mainly characterized by gradual impairment of cognitive function, emotional instability, and behavioral/psychiatric disturbances. MLD can be quite heterogeneous in nature in regard to causative mutations in the *ARSA* gene (12). The ranges of the age of onset and rate of disease progression are broad, which depend on the residual *ARSA* enzymatic activity (13). In general, the process of adult form MLD is slower than that of juvenile and late infantile forms (14). Slow disease progression in the relatively stable and regressive period is a typical feature of adult MLD. The diagnosis of MLD is based on MRI (Symmetrical demyelination of white matter), biochemical (decreased *ARSA* enzymatic activity in leukocytes), and genetic test of the *ARSA* gene (15–17). The patient was characterized by adult onset and the chronic progression of worsening cognitive function. It is speculated that this may be related to the gradual decrease of residual *ARSA* enzymatic activity, and delay in the process of glucosinolates accumulation. According to the age of onset, combined with clinical manifestation, MRI, laboratory examination, nerve conduction, and gene detection, she can be diagnosed as adult MLD.

In MLD, the deficiency of *ARSA* results in sulfatide accumulation in the myelin-producing cells (oligodendrocytes in CNS and Schwann cells in PNS). With the progressive accumulation of sulfatide in cells, the lysosomal-endosomal system becomes dysfunctional, and other secondary pathogenic cascades occur, ultimately resulting in cell apoptosis (18). It causes progressive demyelination in both CNS and PNS, which correlates with the major clinical manifestations of MLD (19). However, as the disease progresses, the symptoms of peripheral neuropathy are gradually masked by the development of spastic tetraparesis and other manifestations of CNS dysfunction. Other PNS symptoms include neurogenic bladder dysfunction, neuropathic pain, and severe foot deformities (20). Therefore, in MLD, the researchers should also be aware of the damage to the PNS as well. The patient had typical brain MRI findings (Symmetrical demyelination of white matter) and a significant slowdown of motor and sensory conduction, which indicated demyelination both in CNS and PNS.

*ARSA* gene mutation could cause the deficiency of *ARSA*, which leads to the development of MLD. It is known that missense, nonsense, and frameshift mutations within the *ARSA* gene can cause MLD (21). Two mutations, namely c.[459 +1G > A] and c.[1277 C > T], occur more frequently in the European population with over 200 mutations reported in MLD patients (22). The c.[459 +1G > A] is frequently found in late infantile patients, and the missense variants c.[1277 C > T] and c.[542 T > G] are usually found in association with an adult or juvenile phenotype (23). The number of samples has been reported to be small, the hot spots have not yet been obtained in China. (24) In addition to pathogenic mutations, an *ARSA* pseudo deficiency (Pd) allele, such as c.[1049 A > G], leads to lower *ARSA* activity, which results in a partial mistargeting of the enzymes (25). The *ARSA*-Pd allele provides sufficient *ARSA* activity to prevent the manifestation of MLD symptoms, even in a homozygous state, or in a compound heterozygous state with an MLD allele. Through Sanger sequencing of the *ARSA* gene, a compound heterozygous mutation can be found, including one reported mutation (11) NM_000487: c.[185_186dupCA], p.(Asp63GlnfsTer18) in exon 1 and a novel mutation NM_000487: c.[154G>T], p.(Gly172Cys), rs74315271.). The mutation of c.[185_186dupCA], p.(Asp63GlnfsTer18), a duplication of CA nucleotides located in exon 1 of c.185_186, leads to a frameshift mutation, causing the amino acid change from Asp to Gln of the first mutation, and then produce a premature termination code. It means that the translated product might have the first 62 amino acids normal, and the subsequent amino acids are aberrantly extended until meeting the poly-adenylation signal. This was an abnormal translation elongation mutation. The mutation was identified as pathogenic according to the American College of Medical Genetics and Genomics (ACMG). It can be also found that the mutation of the patient was inherited from her mother, while her son was the carrier and her daughter was not. Another heterozygous mutation is c.[514G>T] in exon 3, which is a novel missense mutation, resulting in Gly to Cys substitution p.(Gly172Cys). The mutation was identified with uncertain significance according to the ACMG. Prediction of protein function using REVEL, PolyPhen-2, MutationTaster, and GERP+ indicated that the mutation was deleterious. In addition, this study identified that her daughter carried this heterozygous mutation. It was reasonable to assume that the mutation of the patient was inherited from her father, even though her father had passed away. Different from the previous report (11), it is found that her family members who carried one of the two heterozygous mutations did not suffer from such kind of disease. Further research is needed.

According to the aforementioned results of family genetic analysis, there are two heterozygous mutations in this patient's *ARSA* gene, one mutation is inherited from her mother, and the other one is speculated to be from her father. These two mutant genes are in the trans position, forming a compound heterozygous mutation.

As MLD is caused by defective *ARSA*, most therapeutic approaches have tried to correct this biochemical defect, such as enzyme replacement therapy (ERT), bone marrow transplantation (BMT), and gene therapy (GT). Unfortunately, all these treatments could not prevent the progression and improve the prognosis due to the poor permeability of the blood-brain barrier (BBB), which restricts the access of therapeutic compounds during systemic administration and results in the low effectiveness of many therapeutic approaches (26, 27). At present, the treatment of MLD worldwide is still symptomatic and nonspecific. Although this patient has received symptomatic treatment, there is no significant improvement in cognitive function. Therefore, it is particularly urgent to study the treatment methods.

In conclusion, this paper reported a case of a Chinese adult female diagnosed with MLD due to a compound heterozygous mutation in the *ARSA* gene: one known frameshift mutation NM_000487: c.[185_186dupCA], p.(Asp63GlnfsTer18) and one novel missense mutation NM_000487: c.[154G>T], p.(Gly172Cys), rs74315271. Moreover, it can be also found that each family member carries one of the two heterozygous genes except for her dead father and sister. This will provide a critical clue for the prenatal diagnosis of the offspring of this family. This study provided broader insight into critical mutations of *ARSA* in the Chinese population for MLD diagnosis.

## Data availability statement

The datasets presented in this article are not readily available because of ethical and privacy restrictions. Requests to access the datasets should be directed to the corresponding author.

## Ethics statement

The studies involving human participants were reviewed and approved by Ethics Committee of the Second Hospital of Hebei Medical University (Approval Letter No. 2022-P024). The patients/participants provided their written informed consent to participate in this study.

## Author contributions

B-lW, Y-cS, and H-jW conceptualized and designed the study and revised the final manuscript draft. B-lW wrote the first draft of the manuscript. F-lL contributed to data analysis and assisted in the preparation of the manuscript. All authors approved the final version of the manuscript, and agree to be accountable for all aspects of this study.

## Conflict of interest

The authors declare that the research was conducted in the absence of any commercial or financial relationships that could be construed as a potential conflict of interest.

## Publisher's note

All claims expressed in this article are solely those of the authors and do not necessarily represent those of their affiliated organizations, or those of the publisher, the editors and the reviewers. Any product that may be evaluated in this article, or claim that may be made by its manufacturer, is not guaranteed or endorsed by the publisher.
